# Benign Histiocyte-Rich Pseudotumor Developing Postchemotherapy and Mimicking Residual Disease

**DOI:** 10.1155/2020/4674103

**Published:** 2020-07-02

**Authors:** Amrit P. Singh, Glenn P. Murray, Soumya Pandey

**Affiliations:** Department of Pathology, University of Arkansas for Medical Sciences, Little Rock, AR 72205, USA

## Abstract

Histiocyte-rich pseudotumors (HRPT) developing postchemoradiation therapy are a florid response to treatment and reparative change. Although these are benign processes, clinically and radiologically, these may mimic recurrent/relapsed disease. We describe a case of an adult male with history of diffuse large B-cell lymphoma (DLBCL), status postchemoradiation therapy, who developed HRPT at the site of original involvement, mimicking relapse of disease on positron emission tomography/computed tomography (PET/CT) imaging. This is one of the few reported cases of posttreatment HRPT. This entity is important to point out the limitations of PET/CT imaging in patients with lymphomas and metastatic disease and stresses the importance of an excisional biopsy for determining relapse and the need for further treatment.

## 1. Introduction

Chemoradiation therapy is a commonly prescribed regimen for malignant disease [1–3]. Although chemoradiation therapy has improved overall survival rates, many patients present with acute and late toxicities from chemoradiation therapy. Some of the common toxicities include mucositis, hematologic toxicity, neurotoxicity, ototoxicity, and nausea [3]. Rare case reports have been published on sporadic development of HRPT postchemoradiation therapy in patients with lymphomas and solid tumors [4–7]. Clinically, these pseudotumors can present as a slow-growing mass with minimal mass effect or can be an aggressive and locally destructive lesion mimicking malignancy or relapse. Radiologically, these lesions show increased metabolic activity and mimic residual/recurrent disease.

HRPT, also described as inflammatory pseudotumors [4], are a benign inflammatory response and reparative change to therapy. An excisional biopsy aids in definitive diagnosis and excluding residual/recurrent disease. Histologically, these lesions have a xanthogranulomatous appearance composed of numerous vacuolated macrophages, multinucleated giant cells, and a lymphocytic infiltrate in a background of necrosis. Occasionally, cholesterol clefts may be noted, but tumor cells are not present.

Here, we describe such a case of an adult male with history of diffuse large B-cell lymphoma, status postchemoradiation therapy, who presented with a mass at the original site of diagnosis that clinically and radiologically mimicked residual disease. This case highlights the limitations of imaging studies and the importance of an excisional biopsy in reaching a definitive diagnosis to guide further treatment options.

## 2. Case Report

The patient is a 34-year-old nonsmoker Caucasian male with a history of cutaneous DLBCL of the scalp, who was treated with external radiation therapy. The patient was in remission for about 5 years, when he presented with a right neck mass. PET/CT studies showed increased metabolic activity in the right cervical lymph nodes as well as in the spleen. An excisional biopsy of the lymph node confirmed involvement by DLBCL. A staging bone marrow biopsy was performed and was negative for lymphoma.

**Figure 1:**
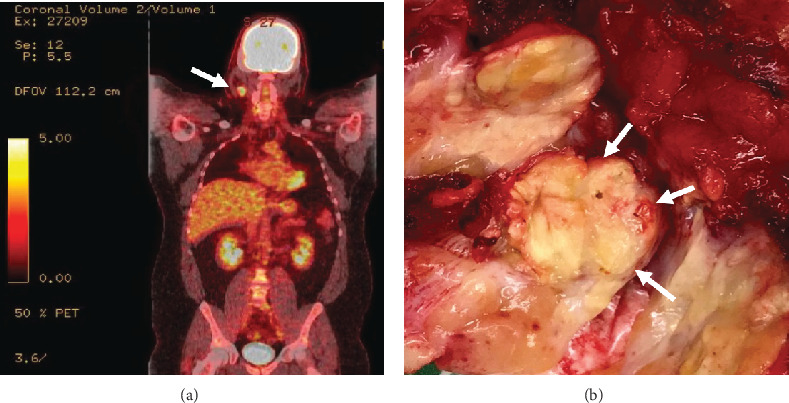
(a) PET scan shows increased uptake in the right cervical lymph node (white arrow). (b) Gross picture of the lymph node cross-section showing tan-yellow nodular areas with focal necrosis.

The patient received cyclophosphamide, doxorubicin, vincristine, and prednisone, plus rituximab (R-CHOP) for 4 months. Post-PET imaging revealed residual activity in the right neck node, and he underwent consolidative radiation therapy. The patient's right cervical lymphadenopathy persisted, and a repeat PET/CT scan performed 6 months later revealed increasing standardized uptake value (SUV) from 3 to 5.4 and ^18^F-fluorodeoxyglucose (FDG) avidity (Figure 1(a)) concerning for residual/refractory disease.

The patient was asymptomatic and denied weight loss, fevers, night sweats, dysphagia, odynophagia, otalgia, pain/tenderness, hoarseness, bleeding, hemoptysis, or cranial neuropathies. An excisional biopsy of the neck mass was performed to rule out residual disease.

Gross examination revealed a soft tan-yellow fibroadipose tissue measuring 6.5 × 3.5 × 2.5 cm. After carefully dissecting through the specimen, one lymph node was identified, measuring 2.5 cm in maximal dimension. The cut surface showed tan-yellow nodular surface (Figure 1(b)). Sections were submitted for histologic examination, flow cytometry analysis, and cytogenetic studies.

**Figure 2:**
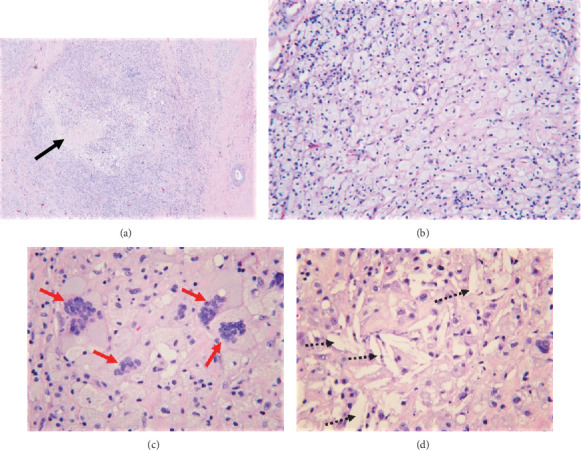
H&E staining (a) shows a diffuse cellular infiltrate with areas of necrosis (black arrow). Higher power at 200x (b) shows the infiltrate composed of numerous foamy macrophages with abundant pink cytoplasm, scattered lymphocytes, (c) multinucleated giant cells (red arrows), and (d) cholesterol clefts (dashed arrows).

**Figure 3:**
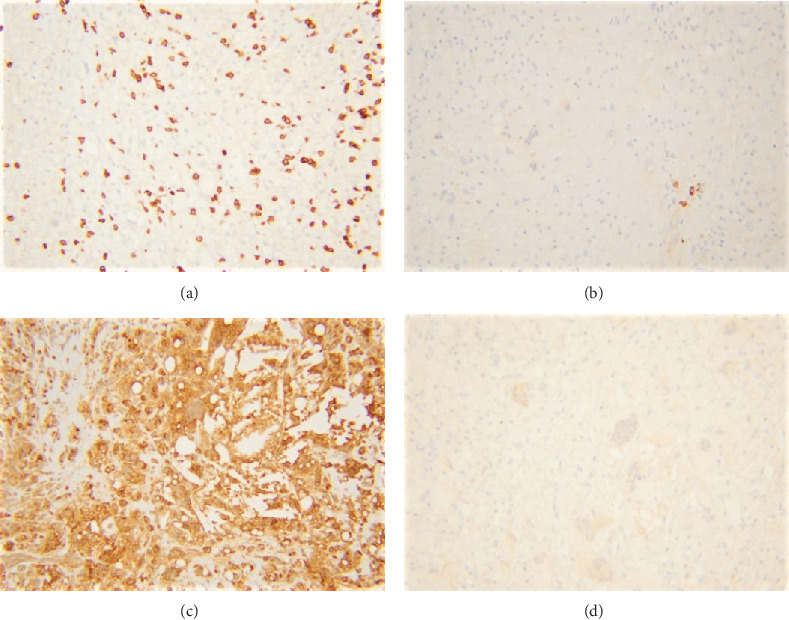
Immunohistochemical stains reveal scattered CD3-positive T-cells (a), rare CD20-positive B-cells (b), and majority of the cells staining positive for CD68 (c). Pancytokeratin stain is negative (d).

Histologic sections revealed fibroadipose tissue with an intense foamy histiocytic infiltrate with numerous giant cells, cholesterol clefts, hyalinizing necrosis, dystrophic calcification, and scattered small lymphocytes (Figures 2(a)–2(d)), morphologically consistent with xanthogranuloma. Mitotic figures were not prominent, and no obvious epithelial cells or carcinomas were found. CD68 immunohistochemical stain confirmed the morphologic findings and marked the diffuse histiocytic infiltrate (Figures 3(a)–3(d)). CD3 and CD20 highlighted only rare small lymphocytes. Pancytokeratin immunostain was negative. Special stains for acid-fast and fungal microorganisms were also negative. There was no evidence of residual/recurrent lymphoma. Flow cytometric analysis could not be performed due to lack of sufficient viable cells. Cytogenetic analysis showed no growth. The overall findings were consistent with HRPT; there was no evidence of a residual lymphoma.

## 3. Discussion

Chemotherapy can induce tumor necrosis and inflammatory response to phagocytize the dead necrotic tissue. Chemokines released from the dying tissue recruit monocytes from the circulation. These monocytes differentiate into activated histiocytes (macrophages) that have phagocytic capabilities. These histiocytes ingest lipid-containing debris and persist as foam cells [5–7]. These histiocytes also release chemokines resulting in recruitment of additional monocytes, thus perpetuating the cycle and resulting in an abundant histiocytic accumulation at the original site. Residual masses are not uncommon in patients with lymphoma after completion of therapy [8, 9], and the clinical differential diagnosis includes persistent lymphoma versus necrotic tumor. CT and ^18^FDG-PET scans are invaluable tools for assessment of treatment response and evaluation of residual lymphoma [8, 10–12]. Although, CT scans are very sensitive for detecting residual masses, they are less helpful in differentiating viable from necrotic lymphoma. ^18^FDG-PET scan offers the advantage as necrotic tissue is not metabolically active [9]. However, ^18^FDG-PET scans are not without limitations, and prior studies have shown false-positive PET scan results, particularly in the first few months after therapy [9, 13, 14]. Postinflammatory changes and abundant histiocytes, as noted in our case, are presumed to cause FDG uptake and false positivity.

Postchemotherapy HRPT have been described in only rare case reports in the past several years [15–17]. This case highlights the limitations of PET/CT scan in evaluation of residual disease and emphasizes the importance of including this entity in the differential diagnosis. Histological confirmation should be obtained, particularly in asymptomatic patients, prior to reinstitution of therapy.

Histologically, these lesions have a xanthogranulomatous appearance composed of numerous vacuolated macrophages, multinucleated giant cells, and a lymphocytic infiltrate in a background of necrosis. Occasionally, cholesterol clefts may be noted, but tumor cells are not present. The most important feature of this entity is an abundance of cytologically benign histiocytes without any nuclear atypia. The mitotic count in these entities is null to very low as this is a result of margination of macrophages and not due to proliferation. If any nuclear atypia or increased mitotic figures are identified, it is important to rule out other neoplasms that can mimic histological features of this entity, such as Follicular Dendritic Cell Sarcoma (FDCS), Langerhans Cell Histiocytosis (LCH), Gaucher's disease, Inflammatory Myofibroblastic Tumor (IMT), and histiocytic sarcoma. Fortunately, these other more worrisome entities can be easily differentiated using appropriate immunohistochemical markers and a good clinical history.

FDCS, specifically the inflammatory pseudotumor-like variant, can mimic HRPT. However, this variant of FDCS has a marked female predominance and often presents with systemic symptoms [18]. Most of the cases are associated with Epstein-Barr Virus (EBV) [19]. Morphologically, cells are more atypical than what were noted in our case. In addition to histiocytic markers (CD163 and CD68), additional stains such as CD21, CD35, CD23, and clusterin [20] can help in differentiating the two entities.

LCH shows predilection for osseous, pulmonary, and integumentary systems, but a minority of patients can present with lymph node involvement [21]. Histologically, they are a clonal proliferation of Langerhans cells [22], cells with abundant, pale eosinophilic cytoplasm, irregular nuclei, prominent nuclear grooves and folds, fine chromatin, and indistinct nucleoli. These most often present as granulomas, foci of necrosis, often surrounded by a rim of eosinophils [23]. While our case showed abundant necrosis, it lacked other characteristic findings of LCH. Additional stains such as HLA-DR, CD1a, S100, Langerin (CD207), and fascin can aid in diagnosis.

Gaucher's disease is more prevalent in Ashkenazi Jews and presents with hepatosplenomegaly, bone lesions, and cytopenias [24]. Histologically, the tissue is infiltrated by ovoid histiocytes, with abundant blue gray lipid-laden cytoplasm that is crinkled or wrinkled paper-like. These cells stain positive for iron, CD68, and PAS [25, 26].

IMT is a proliferation of myofibroblasts with background of plasma cells, eosinophils, histiocytes, and lymphocytes. However, there are prominent/atypical mitotic figures present with atypical polygonal cells with oval nuclei. The cells are positive for smooth muscle actin. Chromosomal translocations leading to activation of the anaplastic lymphoma kinase (ALK) can be detected in approximately 50% of IMTs, particularly those arising in young patients [27, 28].

Histiocytic sarcoma is an aggressive disease associated with B symptoms, lymphadenopathy, and hepatosplenomegaly. The tumor cells are histiocytic in nature, staining positive for CD68, CD163, CD11c, lysozyme, and variable S100 and negative for CD1a, CD13, and CD21. However, the cells are very atypical with variable pleomorphism, irregular nuclei with prominent nucleoli. There is necrosis, giant cells, and mitotic figures easily identified [29].

The cervical mass in our patient lacked the atypical morphological features noted on these more aggressive entities mentioned above. However, if there is any doubt, immunohistochemistry is indispensable to rule out more aggressive and malignant processes.

In conclusion, HRPT is a benign florid response to chemotherapy-induced cell necrosis leading to margination and accumulation of cytologically benign histiocytes in the tissue, which may appear metabolically active on PET-CT. An excisional biopsy should be performed in such cases for definitive diagnosis of residual disease and need for additional therapy.
